# Targeting ECM Disrupts Cancer Progression

**DOI:** 10.3389/fonc.2015.00224

**Published:** 2015-10-20

**Authors:** Freja A. Venning, Lena Wullkopf, Janine T. Erler

**Affiliations:** ^1^Biotech Research and Innovation Centre (BRIC), University of Copenhagen (UCPH), Copenhagen, Denmark

**Keywords:** extracellular matrix, collagen I, hyaluronan, periostin, tenascin C, metastasis

## Abstract

Metastatic complications are responsible for more than 90% of cancer-related deaths. The progression from an isolated tumor to disseminated metastatic disease is a multistep process, with each step involving intricate cross talk between the cancer cells and their non-cellular surroundings, the extracellular matrix (ECM). Many ECM proteins are significantly deregulated during the progression of cancer, causing both biochemical and biomechanical changes that together promote the metastatic cascade. In this review, the influence of several ECM proteins on these multiple steps of cancer spread is summarized. In addition, we highlight the promising (pre-)clinical data showing benefits of targeting these ECM macromolecules to prevent cancer progression.

## Introduction

Metastases cause more than 90% of cancer patient death (1). The spread of the tumor cells to secondary sites of the body is a complex process involving reciprocal interaction between tumor cells and their microenvironment (2). Metastases are the result of a series of complex processes, including the escape from the primary tumor, the invasion into adjacent tissue, hematogenous or lymphatic spread, the establishment of micrometastases, and the final outgrowth and colonization at the distant site of the body (3). Today, it is becoming widely accepted that the tumor microenvironment crucially affects cancer progression. The tumor microenvironment consists of not only the cancer cells, all non-malignant cell types such as immune cells, fibroblasts, pericytes, endothelial cells, adipocytes, and mesenchymal stem cells, but also the interstitial fluids and the extracellular matrix (ECM). This review will summarize how ECM composition and structure, at both the primary and the secondary site, are key factors for a successful metastatic spread.

The ECM is a complex meshwork of macromolecules secreted by the different cell types of a tissue, made up of both proteins and proteoglycans (PGs) with covalently attached sugar chains, glycosaminoglycans (GAGs). Besides providing the structural support of an organ, the ECM is instrumental in modulating cell functions. Beyond direct interaction with cellular signaling receptors, the network of macromolecules also functions as a reservoir for growth factors or signaling molecules, thus influencing cellular behavior indirectly (4). Together ECM signaling can be involved in proliferation, migration, invasion, the onset of angiogenesis, or the resistance to apoptotic stimuli. In addition, ECM proteins can work as an anchor and promote cellular adhesion. Moreover, fibers of ECM proteins such as collagens can build migration tracks for the tumor cells. At the same time, the ECM can function as a barrier blocking, e.g., the penetration of immune cells into the tumor, or it can create a high interstitial fluid pressure (IFP) preventing the perfusion of drugs, which facilitates chemoresistance.

**Figure 1:**
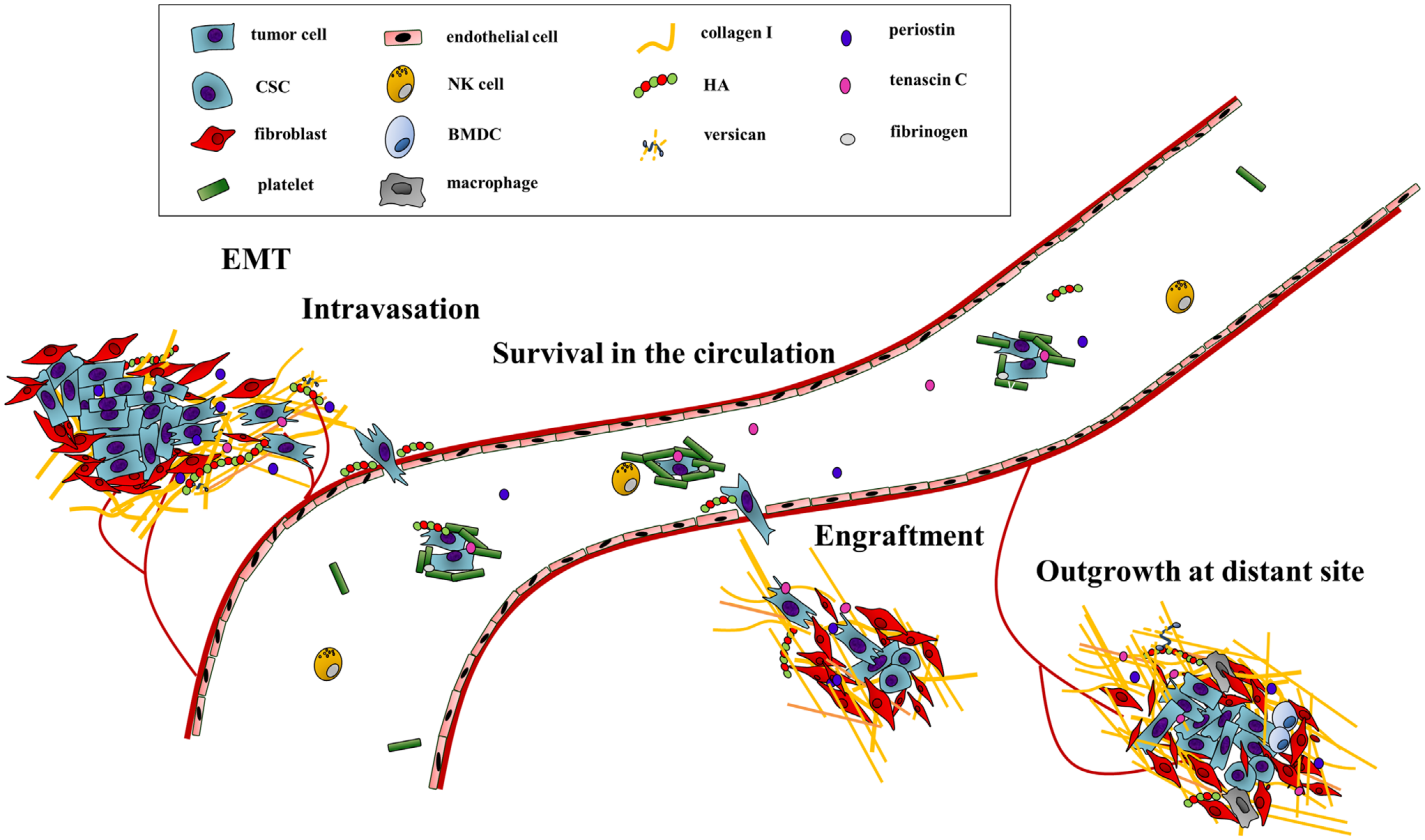
**The ECM drives the progression of cancer cells along the metastatic cascade**. The metastatic cascade is composed of multiple complex processes, which are critically influenced by ECM components. First, ECM-regulated signaling pathways increase cancer cell motility and promote the egress from the primary tumor. In addition, the stability of the endothelial cell barrier is critically regulated by HA, thus influencing intra- and extravasation efficacy of cancer cells. The survival in the circulation system is also directly and indirectly modulated by ECM components as they function as physical shields as well as attractants for platelets. Through deposition and modification of ECM components at distant sites, the initial engraftment and final colonization of cancer are enhanced. Hereby, biochemical as well as biomechanical cues of the ECM promote metastatic outgrowth.

In this review, we will first briefly introduce a handful of well-studied ECM components and then describe their contribution to the steps of the metastatic cascade (Figure 1). On the protein side, the focus will be on the matricellular proteins periostin and tenascin C as well as on the fibrillar collagen I. We will also look into the role played by the unique GAG hyaluronan (HA) and its often partner-in-crime versican. Second, we will discuss therapeutic approaches that either directly target the ECM components or their modification.

## Key Players

### Periostin

Periostin, also known as osteoblast-specific factor 2 (OSF-2), is a secreted N-glycoprotein that was identified as a cell adhesion protein in a mouse osteoblast cell line (5). This matricellular protein was shown to interact with itself and other ECM proteins as collagen I, fibronectin, and tenascin C (6). Initially, periostin was associated to cancer as high expression levels were found in patient samples of common solid tumor types such as breast, colon, lung, and pancreatic cancer, as well as melanoma (7–11). The expression level correlates with tumor progression and was shown to be especially elevated in the secondary sites in 75% of the lymph node metastases of breast cancer patients (12). Besides, periostin can be found in the serum of patients with advanced metastatic disease (13–15). Therefore, periostin qualifies as a tumor marker in the clinic, especially for advanced breast cancer.

### Tenascin C

The tenascin family has five members named tenascin C, R, W, X, and Y (16). The secreted glycoproteins bind to a great variety of proteins as periostin, fibronectin, integrins, and several collagens. This review will focus on the role of tenascin C in advanced tumorigenesis. Tenascin C expression is restricted to connective tissues and interestingly stem cell niches in adult tissues but is very prominent in tumor tissue (17–22). Stromal expression was particularly observed in late-stage tumors, with a particular strong staining at the zone of tumor–stroma interaction (17, 18, 23). Elevated serum levels make tenascin C suitable for clinical monitoring of the most common cancer types (24–26).

### Hyaluronan

Hyaluronan is an important GAG in the ECM of many adult tissues, and an increase in HA deposition is seen in many solid cancers, particularly of the prostate, pancreas, breast, and bladder (27), often correlating to a poor prognosis (28). HA is the only GAG composed of unsulfated disaccharides (d-glucuronic acid and *N*-acetylglucosamine), and it is never covalently attached to a proteoglycan core protein (29). HA is synthesized by hyaluronan synthases (HAS), which produce high-molecular-weight HA (HMW-HA) above 1000 kDa (30), which can be degraded into low-molecular-weight (LMW)-HA and even smaller oligo-HA by either hyaluronidases (HYAL-2, -2, -3, and PH20) or reactive oxygen species (ROS) (31). Interest in HA’s role in cancer progression was recently rekindled by the seminal finding that the extremely large HMW-HA produced by the naked mole rat (five times larger than human HMW-HA) is essential for its remarkable resistance to cancer development (32–34).

### Versican

Versican is a large chondroitin sulfate proteoglycan (CSPG) that is present around cells in most healthy tissue in low amounts and upregulated in malignancies of, e.g., the breast, colon, prostate, lung, and ovaries, among others (35). Versican binds to a number of other ECM components, notably HA, contributing to the formation of a biomechanically active pericellular matrix that affects the proliferation, adhesion, and motility of cells (36–39). Clinically, increased versican expression correlates to a decreased progression-free survival in prostate cancer (40), increased relapse in breast cancer (41), advanced disease and lymph node metastasis in adenocarcinomas of the lung (42), and is also diagnostically relevant in other cancers [reviewed in Ref. (35)].

### Collagen I

Collagen I is the main fibrillar collagen in the ECM, providing tensile strength to the tissue and limiting its distensibility. Collagen I fibrils in normal tissue are made up of processed heterotrimers of two col1α1 and one col1α2, which self-assemble in the extracellular space into fibrils. The fibrils are cross-linked by enzymes of the lysyl oxidase (LOX) family (43), forming larger mechanotransductive fibers that increase the density and rigidity of the tissue (44). Increased mammographic density correlates to an increase in collagen deposition (45) and more importantly to an increased risk of developing breast cancer (46). Abnormally large collagen deposition is the most well-documented ECM alteration in many tumor types, and collagen deposition has been causally linked to an increase in mammary tumor and metastasis incidence (47).

## Escaping the Primary Tumor: EMT and Intravasation

**Figure 2:**
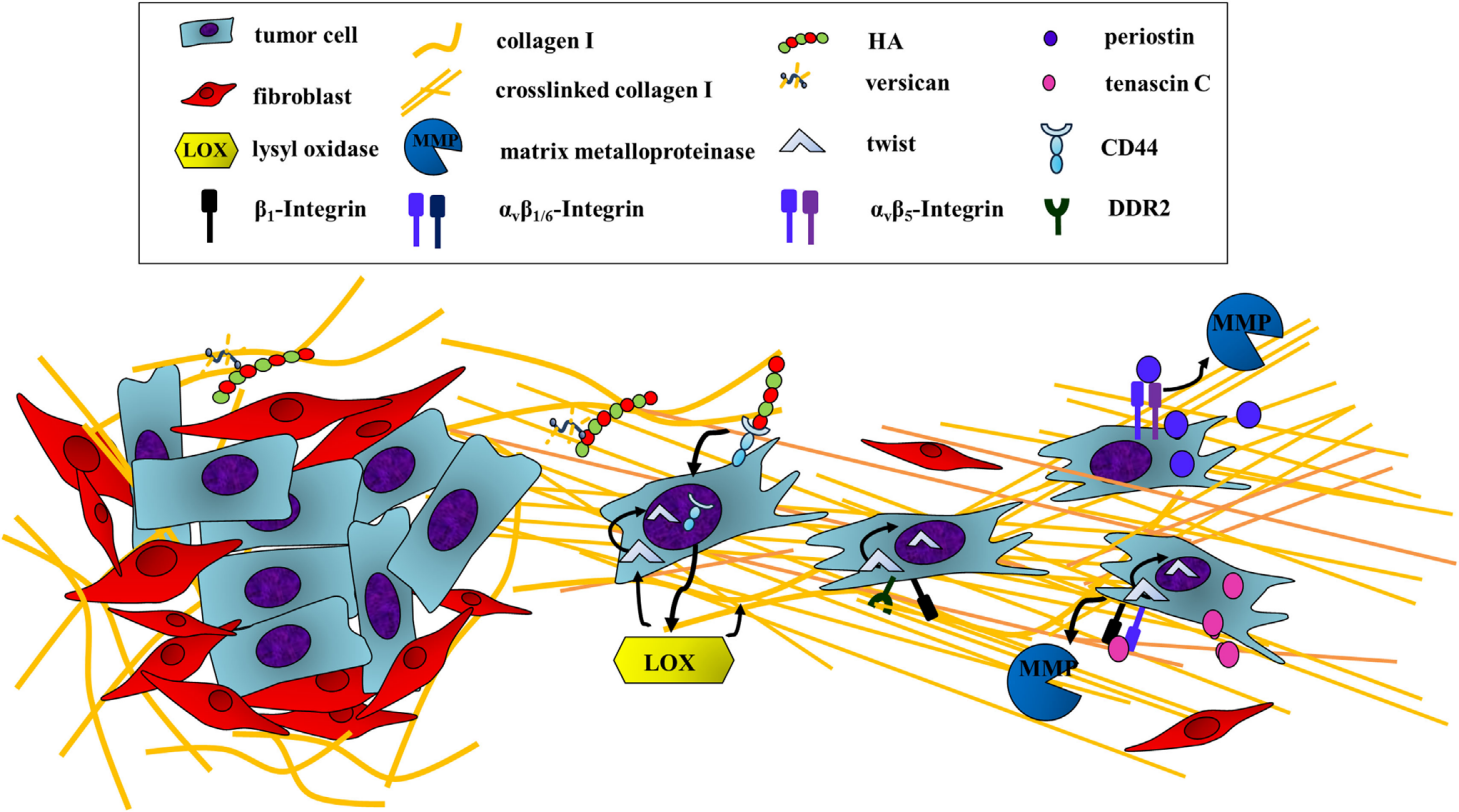
**Escaping the primary tumor: ECM components induce cancer cell motility**. In order to leave the primary tumor, cancer cells undergo epithelial-to-mesenchymal transition (EMT), which can be induced by ECM proteins and GAGs activating receptor-mediated signaling pathways. First, HA binding to CD44 on tumor cells induces EMT through the translocation of the receptor to the nucleus. CD44 is then able to induce a transcriptional upregulation of LOX. LOX promotes EMT by regulating the EMT transcription factor TWIST-1 through two mechanisms. First, LOX is able to activate the expression of TWIST-1. In addition, the activity of TWIST-1 is indirectly enhanced by LOX through association of cross-linked collagen I with integrin β1 and DDR2, which promotes the nuclear translocation of cytoplasmatic-bound TWIST-1. Besides, tenascin C also influences the transcriptional regulation of EMT indicated by the downregulation of E-cadherin and a simultaneous upregulation of vimentin as well as several MMPs. Periostin also enhances MMP expression, thus inducing EMT through binding to tumor cell α_v_β_5_-integrin.

The first step of tumor dissemination is for the cancer cells to break free from the confinements of the primary tumor. They have to acquire the ability to move and invade through the basement membrane as well as the walls of vessels of the blood stream or the lymphatic system, a process called intravasation (48). In order to detach from the primary site, some tumor cells undergo epithelial–mesenchymal transition (EMT). EMT is defined by a simultaneous downregulation of epithelial proteins such as E-cadherin and an upregulation of mesenchymal proteins such as N-cadherin and vimentin leading to the loss of cell–cell contacts and an increase in cell motility (49). Many ECM proteins are associated with the induction of EMT by activating receptor-mediated signaling cascades (Figure 2) (50, 51).

First, the production of the glycosaminoglycan HA has been demonstrated to induce EMT in both normal and transformed epithelial cells *in vitro* [reviewed in Ref. (31) and references therein]. *In vivo*, accumulation of HA in pancreatic and mammary tumor models is associated with loss of E-cadherin and nuclear translocation of β-catenin (52, 53), both hallmarks of EMT. However, overproduction of HA is not in itself enough to create an invasive phenotype, on its own it actually decreases cell motility and tumorigenesis (54). However, if the general turnover of HA is increased due to high levels of both HA synthases and hyaluronidases, i.e., increased levels of LMW-HA, then this leads to an increase in cell motility *in vitro*. This is mirrored *in vivo* by the appearance of spontaneous lymph node metastasis in a mouse model of pancreatic cancer with increased HA-turnover (54). One mechanism of HA-induced EMT involves binding to the cellular receptor CD44, which then translocates to the nucleus and by binding to the promoter leads to the upregulation of LOX. Next, LOX catalytic activity is, in a so far undetermined manner, necessary for the expression of the EMT transcription factor TWIST-1 (Figure 2) (55).

The formation of an HA-rich pericellular matrix is important for proliferation and motility of normal mesenchymal cells (36), a phenomenon cancer cells also utilize (37–39, 56, 57). Studies of ovarian cancer cells and leiomyosarcoma cells have showed that versican is necessary for the formation of this HA-rich pericellular matrix (38, 39). Knockdown of versican expression in ovarian cancer cells decreased their motility *in vitro* and more interestingly also their ability to form experimental metastases after injection into the peritoneal cavity (58).

Besides its role in general motility, HA has a particular important function in the process of intravasation. HA regulates blood vessel integrity, with HMW-HA and LMW-HA degradation products playing opposite roles. HMW-HA promotes endothelial cell barrier function through several mechanisms while LMW-HA disrupts it (59–61). Furthermore, LMW-HA is also angiogenic (62), so the production of LMW-HA fragments in the tumor microenvironment can thus compromise the tumor vessel integrity and promote angiogenesis, making it easier for cancer cells to intravasate and continue the metastatic process.

Studies of both patient material and mouse models of cancer have shown that the deposition of a collagen-rich matrix is linked to tumor progression and metastasis (47). Collagen I is indeed intricately involved in the induction and maintenance of EMT and an invasive phenotype. *In vitro* studies have shown that interaction between collagen I and integrin β1 leads to destabilization of the E-cadherin–beta-catenin complex and also to upregulation of N-cadherin (63, 64). Recently, it has been reported that inhibition of collagen synthesis in human MDA-MB231 breast cancer xenografts leads to a decrease in local invasion into the surrounding adipose tissue and to a decrease in metastasis to both the draining lymph nodes and lungs (65, 66). The level of circulating tumor cells was decreased in mice where collagen synthesis was inhibited, further demonstrating that the collagen content of the primary tumor is important for generating invasive cancer cells capable of intravasation (65).

Changes in the collagen matrix in tumors also provide altered biomechanical cues to tumor cells. Enzymes of the LOX family catalyze the cross-linking of collagens and elastin, increasing the tissue stiffness (43). LOX and LOX family members are frequently overexpressed in cancers (43), and their collagen cross-linking activity has been proven to promote tumor progression through increased integrin signaling (67–70). Additionally, the tissue stiffness is essential for determining the cellular response to the potent EMT inducer TGF-β, as EMT signaling is only induced in cells residing in a stiff tissue, with apoptosis being the go-to program for cells in a soft ECM (71). The mechanism behind this stiffness-regulated switch was decoded recently, showing that the transcription factor TWIST-1, which is essential for EMT, translocates to the nucleus due to stiffness-induced release from its cytoplasmic anchor G3BP2 (Figure 2) (72).

It is not only the amount and stiffness of the collagen network that is important; the orientation of collagen fibers also appears to be central to the progression of cancer. Through intravital imaging of tumors several studies have shown that the organization of collagen into straight, aligned fibers promotes cell invasion along these fibers (73). In breast cancer, the orientation of these collagen fibers in relation to the tumor is an independent prognostic indicator, with fibers aligned perpendicular to the tumor correlating to a poor disease-specific and disease-free survival (hazard ratio >3) (74). Molecular evidence for this clinical correlation was provided by Zhang et al. in a mouse model of breast cancer, showing that binding of collagen I to the cell surface receptor discoidin domain receptor 2 (DDR2), a receptor tyrosine kinase, leads to the formation of collagen fibers oriented perpendicular to the surface of the tumor, facilitating cells invading out along these (75). Furthermore, DDR2 is upregulated in cells undergoing EMT, and binding of collagen I to DDR2 further sustained the EMT transcriptional program, providing a positive feedback loop (Figure 2) (75).

Bornstein et al. introduced the term matricellular proteins to describe the family of non-structural extracellular proteins (76), among which periostin and tenascin C are especially important for the metastatic cascade. During the last decade, increasing evidence has amassed for the functional importance of periostin in the initiation of the metastatic cascade via the induction of EMT. First, overexpression of the periostin in 293T cells led to an augmented expression of vimentin correlating with a mesenchymal morphology as well as an increase in migration and invasion. These effects were dependent on α_v_β_5_-integrin binding to periostin (Figure 2) (77). In addition, these cells also showed an increase in expression of the matrix metalloproteinase MMP-9. Proteases degrade the ECM in the cell’s surroundings, paving the way for the cells through the dense environment (78). Moreover, elevated levels of periostin mRNA and protein were revealed at the invasive front of immortalized esophageal cells *in vitro* and *in vivo*. Overexpression of periostin in these cells increased their ability to move while depriving them of the matricellular protein reduced the invasive potential significantly (79). The same was reported in oral squamous cell carcinomas, where overexpression of periostin facilitated motility and invasiveness *in vitro* as well as in an orthotopic mouse model (80).

A further mediator of cell motility is tenascin C. By binding to either integrin α_v_β_1_ or α_v_β_6_, recombinant tenascin C induced a change in the morphology of the breast cancer cell line MCF-7 to a more mesenchymal phenotype (Figure 2) (81). Besides, Tavazoi et al. were able to diminish the invasive potential of a metastatic breast cancer cell line by knocking down tenascin C expression. These cells were also deprived of the ability to form lung metastasis *in vivo* (82). Moreover, tenascin C induces expression of several matrix metalloproteinases (Figure 2) (83), again linking it to the onset of invasion (84). Both observations are in accordance with the striking expression of tenascin C seen at sites of epithelial–mesenchymal interactions during development (85) and at the invasive front of human breast cancer samples (86). Oskarsson et al. could even demonstrate a deposition of tenascin C at the margin of lung metastases of both mice injected with the breast cancer cell line MDA231-LM2 and breast cancer patient samples (23).

## A Journey in the Blood: Resisting Mechanical Forces and Immunoescape

**Figure 3:**
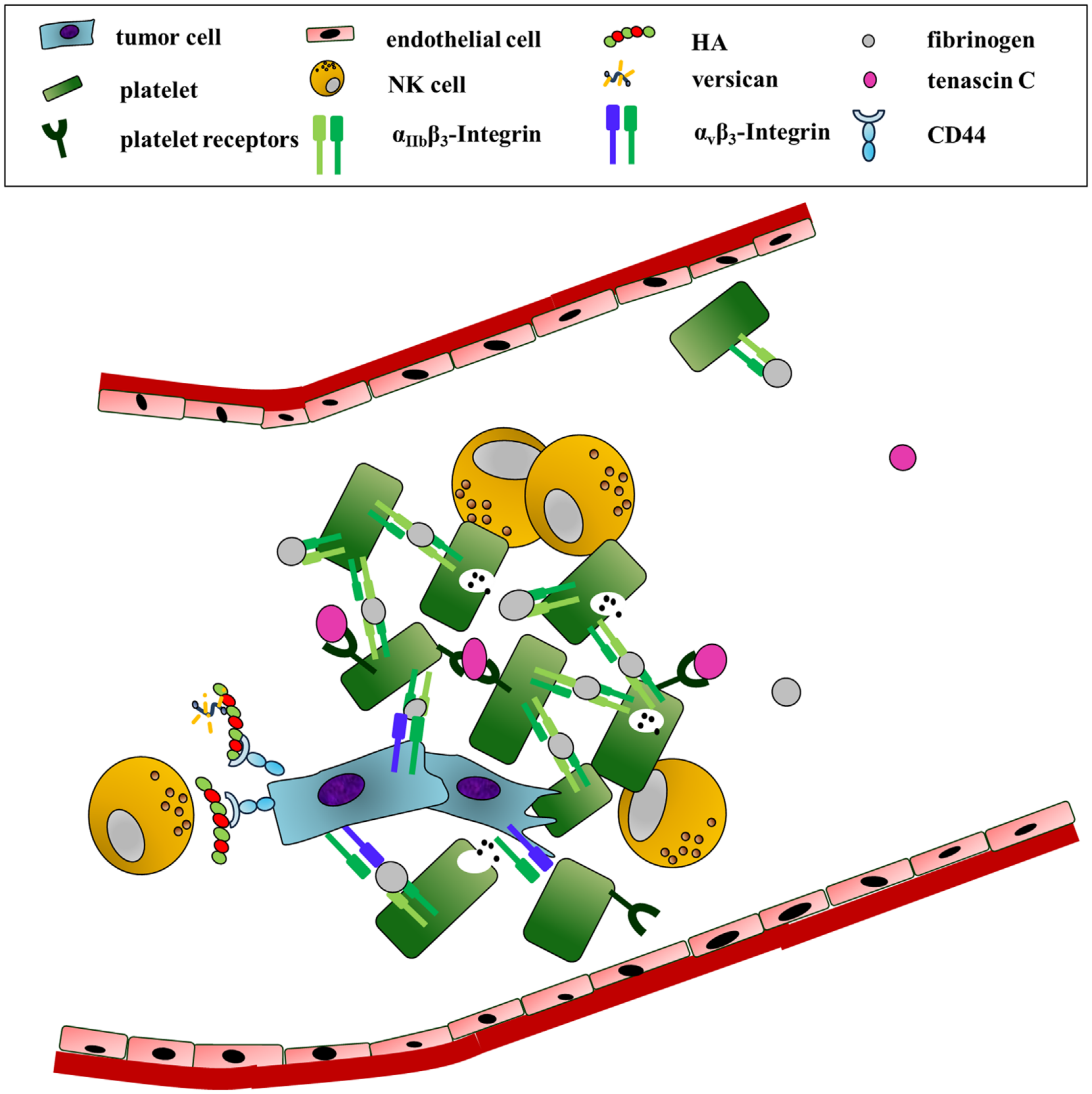
**Survival in the blood: ECM components as physical shields and mediators of platelet recruitment**. In order to survive the high shear forces and patrolling NK-cells in the blood circulation, tumor cells interact with platelets. This association is mainly initiated by fibrinogen. Fibrinogen binding to tumor-expressed α_v_β_3_ and platelet-derived integrin α_IIb_β_3_ induces the formation of cancer-cell–fibrinogen–platelet complexes. In addition, tenascin C association with integrin α_2_β_1_ or the glycoprotein Ib-IX complex enhances platelet adhesion and activation. An HA-rich pericellular matrix further shields the cancer cells from NK-cell attack.

Once tumor cells manage to evade the constraints of the primary tumor and successfully invade the lymphatic or hematologic system, they are exposed to a hostile and deadly environment. As the transition to distant organs depends on the blood system, we will focus on the tumor cell survival in the blood circulation. Here, cancer cells have to resist shear forces and turbulence. In addition, they have to escape the immune surveillance, especially natural killer (NK) cells. The main protective mechanism for tumor cells is an interaction with platelets that work as a shield against immune cell lysis and mechanical stress (87). The most important mediator is the acute phase protein fibrinogen. Fibrinogen is mainly secreted by hepatocytes and megakaryocytes but can also derive from tumor cells (88). Although being primarily a plasma protein, fibrinogen has many roles as an ECM protein in cancer (89). Fibrinogen can bind to thrombocyte receptors inducing platelet adhesion. Both fibrinogen and especially its protease converted form fibrin can provoke α_v_β_3_-integrin and α_IIb_β_3_-mediated cancer-cell–fibrin(ogen)–platelet complexes (Figure 3) (90, 91). The promoting role of fibrinogen and platelets in hematogenous metastasis was confirmed by a striking decrease in melanoma cells colonizing the lung in either fibrinogen knockout or platelet deprived, protease-activated thrombin receptor 4 (PAR4) knockout animals (92–94).

However, other ECM proteins can also promote the recruitment, binding, and activation of platelets (95, 96). Platelets adhered efficiently to tenascin C in static *in vitro* assays as well as under dynamic flow. Tenascin C also enhanced platelet activation (97). Although not specifically shown in cancer, tenascin C therefore could indirectly support tumor survival in the blood by promoting platelet recruitment and activation (Figure 3).

An HA-rich pericellular matrix could be another type of shield the cancer cells employ to ward off NK cells. *In vitro* studies have shown that HA effectively keeps lymphocytes such as NK cells from getting in close contact with the cancer cells, preventing them from killing the cancer cells (Figure 3) (57, 98). Thus, the ability of the cancer cell to produce such a pericellular matrix, requiring CD44, HA, and versican (or aggrecan), could add to the chances of survival in the circulation.

## Finding a New Home: The Challenge to Extravasate and Survive

Seeding of cancer cells in a secondary organ requires the extravasation from the circulation, initial adherence, and the initiation of proliferation under the unpermissive conditions of the secondary sites.

**Figure 4:**
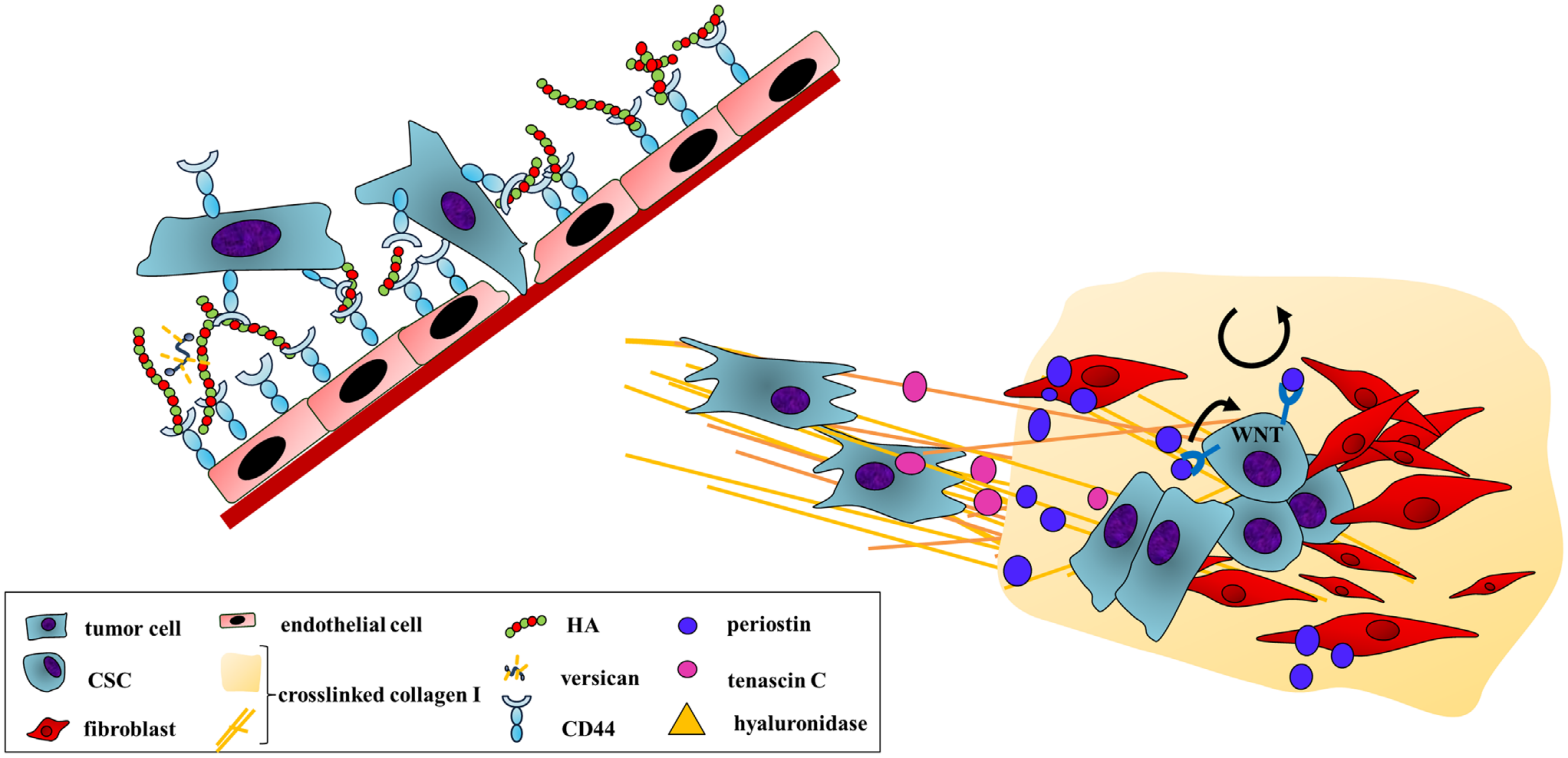
**A distant home: tumor cell extravasation and engraftment at secondary sites are enhanced by ECM secretion and remodeling**. Extravasation requires cancer cell adhesion to the vessel wall and an invasion into the foreign tissue. HA mediates an initial adherence as both endothelial and cancer cells bind to the GAG through CD44. A subsequent secretion of hyaluronidase breaks down HMW-HA. The local increase of LMW-HA disrupts the endothelial cell barrier supporting transendothelial migration of the cancer cells. After arrival at a distant site, cancer cells are exposed to a foreign environment. To allow survival under these different conditions, cancer cells secrete ECM components and ECM-remodeling enzymes to favor the engraftment at the foreign site. An example is tumor-derived LOX altering collagen cross-linking at pre-metastatic sites. Tumor cell-secreted tenascin C also enhances the establishment of micrometastases. However, tumor cells also induce stromal cells to produce cancer-promoting ECM proteins, creating a more permissive environment. Here, stromal periostin improves cancer cells adhesion by binding to αvβ5-integrin. In addition, periostin supports the self-renewal and proliferation of CSCs through the activation of the WNT signaling pathway, which may enhance outgrowth of metastases.

In order to leave the circulation, cancer cells need to adhere to the endothelium and push through into the tissue. Endothelial cells have a thick, pericellular matrix rich in HA, the glycocalyx, and tumor cells can interact with this through CD44 to initiate adhesion (Figure 4) (99). The importance of cancer cell interaction with HA on endothelial cells in the process of extravasation is backed by a recent *in vivo* study, which showed that knockdown of the HA receptor CD44 in MDA-MB-231 breast cancer cells drastically decreased the number of experimental metastases in an intracardiac dissemination model (100).

Cancer cells may also construct a pericellular matrix rich in HA (57), and the reciprocal interaction of this with receptors on endothelial cells also appears to be important for adhesion (101–104) and the following transmigration into tissue (103). Once cancer cells have adhered to the endothelium, it is possible that tumor cell-secreted hyaluronidases create a local increase in LMW-HA, from the breakdown of the glycocalyx, which then in turn can lead to the disruption of the endothelial barrier, opening up the door for the cancer cell.

Hirose et al. recently showed that the levels of circulating LMW-HA in the blood is an important factor for melanoma cell adhesion to endothelial cells. Increased serum levels of LMW-HA, created by blocking the HA receptor for endocytosis (HARE/Stab2) found in sinusoidal endothelium in the liver, bone marrow, and lymph nodes, prevented B16F10 melanoma cells from binding to endothelial cells and producing lung foci in tail-vein injected mice (105). Strikingly, elevated LMW-HA in serum also prevented spontaneous lung and lymph node metastasis of orthotopically implanted human MDA-MB-231 and mouse 4T1 breast cancer cells (105). Similarly, Simpson et al. have reported that prostate cancer cells bind to sinusoidal endothelial cells through HARE/Stab2 and that blocking HARE *in vivo* via an antibody completely prevented spontaneous lymph node metastasis in an orthotopic prostate cancer model (104). Two cooperating mechanisms may be at play here. Blocking HARE or saturating it with HA can prevent direct binding of cancer cells to HARE+ cells through their HA-rich pericellular matrix and simultaneously the elevated blood levels of HA, created by lack of HARE-mediated clearance, might also saturate CD44 on the cancer cells, preventing them from utilizing this previously demonstrated important factor for extravasation. Yet, a recent study has found that elevated plasma levels of LMW-HA correlate with lymph node metastasis in breast cancer patients (106). An explanation for this discrepancy could be the fact that serum levels of LMW-HA that prevent extravasation and metastasis in mice were more than fourfold higher than normal serum levels, and the difference in serum LMW-HA that separated metastatic vs. non-metastatic patients was less than twofold. This suggests that the modest increase in serum LMW-HA seen in metastatic breast cancer patients, while prognostically indicative, confers no protective advantage to offset the pro-metastatic benefits of increased intra-tumoral LMW-HA.

Lysyl oxidase, LOXL2, or LOXL4 expression in the primary breast tumor leads to pre-metastatic deposition of collagen I in the lungs of mice (Figure 4) (65, 107), favoring the formation of metastases in the lungs (65, 107–109). Furthermore, LOX-mediated cross-linking of collagen I increases the fibrotic response in lungs and livers of mice and helps form a favorable metastatic niche in these organs. Mice with fibrotic lungs and livers had an increased number of spontaneous 4T1 breast cancer metastases to these organs, which was abrogated by blocking LOX activity (110).

Periostin also showed a proadhesive effect on 293T cells, which was αvβ5-integrin-dependent (Figure 4) (77). This is concurrent with the identification of periostin as one of the key components of the so-called metastatic niche (111). In order to convert the new unfavorable surrounding into a more permissive environment, tumor cells secrete factors before and upon arrival at the distant site. Furthermore, they induce stromal cells to produce cancer-promoting extracellular proteins (112). Contié et al. reported a prominent expression of periostin in bone metastasis of tail vein-injected MDA-B02 breast cancer cells (15). Malanchi et al. confirmed this result in the polyomavirus middle T antigen (PyMT) mouse model for spontaneous breast cancer. Here, a deposition of periostin was revealed not only in the primary breast tumor but also even more pronounced in the lung metastasis. By combining the breast cancer model with periostin null mice, the authors could reveal that stromal periostin supports the survival and proliferation of cancer stem cells (CSCs, CD90^+^, and CD24^+^) through the activation of WNT signaling (Figure 4) (12). CSCs have been implicated in the establishment of new micrometastases that then subsequently grow out into macroscopic secondary tumors (113). The reduction in number of metastasis in periostin knockout mice to less than 10% of the control, with a lower number of CSCs and abrogated WNT signaling, affirms this concept (12).

The initial engraftment of tumor cells at secondary sites is also critically influenced by tenascin C (Figure 4). In depriving a human metastatic breast cancer cell line of tenascin C, Oskarsson et al. proved the need of tumor-secreted tenascin C for the establishment of micrometastasis. The tenascin C knockdown resulted in a 90% inhibition of lung colonization in either experimental or spontaneous lung metastases of these cells. Immunohistochemistry probing for the apoptotic marker caspase-3 revealed that tumor cell survival is dependent on tenascin C. In order to specify the effect of tumor endogenous protein production, Oskarsson et al. used an inducible knockdown model narrowing down the time frame for the dependency on tumor-derived tenascin C. Interestingly, depriving the breast cancer cells of tenascin C only affected the outgrowth of metastases when they reached a certain size (23). In pancreatic cancer, ectopic tenascin C expression in RIP-Taq2 mice significantly increased the establishment of micrometastases, whereas a tenascin C knockout reduced tumor cell engraftment in the lungs (22).

## Making Yourself at Home: Macroscopic Outgrowth in an Inhospitable Environment

The low efficiency of engrafted cancer cells to establish bona fide macroscopic metastases indicates that the microenvironment of secondary sites is generally unsupportive for tumor cells. In order to convert an unfavorable surrounding of a distant site into a more permissive environment, which allows macroscopic lesions to develop, tumor cells secrete factors before and upon arrival at the distant site. Furthermore, they induce stromal cells to produce cancer-promoting extracellular proteins (112).

Collagen deposition in the metastases is very important for the progression into larger lesions. Extravasated MDA-MB-231 cells with a knockdown of prolyl-4-hydroxylases, an enzyme essential for correct collagen biosynthesis, were able to survive but formed smaller lung metastases than their wild-type counterparts (65, 66). In line with this, successful colonization of draining lymph nodes by MDA-MB-231 xenografts was accompanied by an increase in collagen deposition in the lymph nodes (114). Additionally, mice with collagen-rich fibrotic lungs and livers tended to develop larger breast cancer metastases (110).

**Figure 5:**
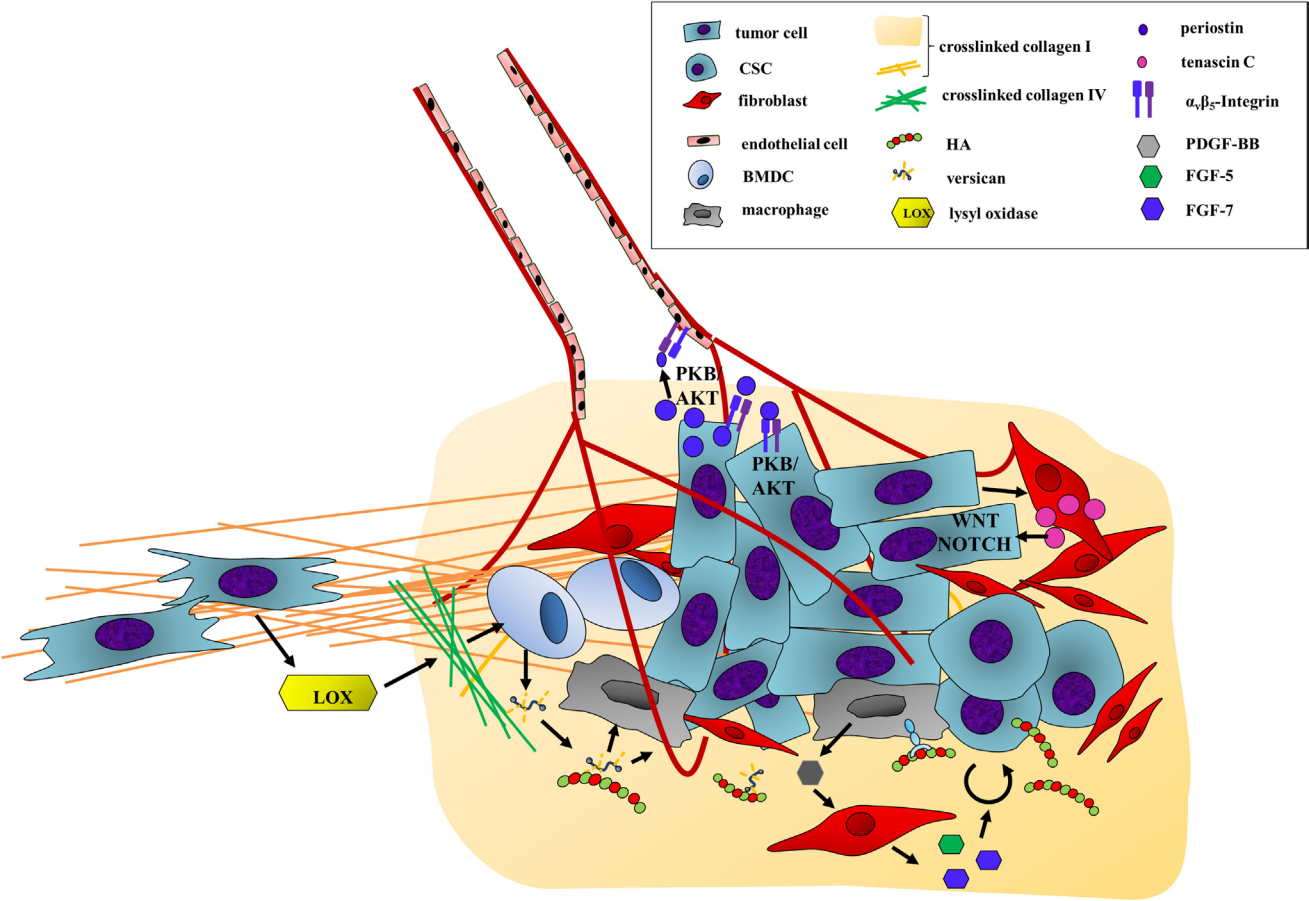
**Final colonization: ECM deposition and remodeling facilitate the outgrowth of metastases**. The final outgrowth of macrometastases is influenced by the deposition, remodeling, and signaling of ECM components. An example is the extensive secretion and alignment of collagen I fibers promoting the final colonization of cancer cells at a distant site. In addition, tumor-secreted LOX cross-links collagen IV, thereby attracting BMDCs. These cells indirectly enhance CSC survival and renewal. BMDC-secreted versican associates with HA. The complex can bind CD44 of macrophages activating their production of PDGF-BB. PDGF-BB in turn mediates other stromal cell secretion of FGF, which finally stimulates the proliferation of CSCs. In addition, versican also induces angiogenesis. An increase in angiogenic activity is also mediated by periostin. Tumor-secreted periostin binds integrin α_v_β_3_ of endothelial and tumor cells. This activates PKB/AKT signaling, promoting cell survival enhancing angiogenesis as well as tumor cell outgrowth. Another mechanism supporting tumor cell survival is the induction of stromal tenascin C secretion. Tenascin C can activate WNT and NOTCH signaling pathways ensuring tumor cell viability under unpermissive conditions.

The recruitment of bone marrow-derived cells (BMDCs) is another key step in the formation of the metastatic niche (115). LOX-mediated cross-linking of collagen IV recruits CD11b^+^ BMDC to the metastatic niche in the lungs (107, 109), and these CD11b^+^ cells further remodel the ECM into a favorable home for extravasating cancer cells, e.g., by laying down the proteoglycan versican (Figure 5) (116). Inhibiting versican production by CD11b^+^ BDMCs radically decreased the burden of lung metastases in a mouse model of spontaneous breast cancer, specifically preventing the progression from micrometastases to macrometastases (116). Previously, versican had been shown to promote mesenchymal to epithelial transition (MET) in fibroblasts *in vitro* (117). In the study by Gao et al., versican also promoted MET *in vivo*, changing the phenotype of the intravasated cancer cells from migratory and slowly proliferative to more adhesive and proliferative (116). Versican could also contribute to the growth of metastases by interacting with HA and forming complexes that can recruit macrophages and induce angiogenesis (53, 118), further molding the secondary site into a supportive home (Figure 5).

Cancer cell-produced HA and the accumulation of HA in experimental bone lesions of MDA-MB-231 breast cancer cells appear to be important for the growth of the lesions (119). One mechanism for HA-induced bone lesion growth was deciphered by Okuda et al., showing that CSCs (CD24^−^, CD44^+^, EpCAM^+^) from bone tropic MDA-MB-231-BoM depend on autologous HA synthesis to survive and renew in the metastatic bone niche. CSC-produced HA interacts with CD44 on macrophages, activating them to produce the growth factor PDGF-BB, which in turn activates other stromal cells to produce FGF7 and FGF9 that stimulate CSC proliferation and self-renewal (Figure 5) (103). This places HA at a central position in the development of a favorable metastatic niche that permits secondary tumor development.

While Malanchi et al. (12) reported an essential role of stromal periostin in the establishment of micrometastases, an earlier study revealed a role of tumor-endogenous periostin in the final step of colon cancer colonization of the liver. Colon cancer cells overexpressing periostin showed a strong increase in hepatic metastases in an experimental metastasis model. Yet, while an equal amount of micrometastases was detected in the first days after intraportal injection, the further outgrowth of these cells was dependent on tumor cell-derived periostin. Tumor cell survival was promoted as periostin binds to α_v_β_3_-integrin, enhancing the PKB/AKT pathway at secondary sites (Figure 5). Besides, the authors linked periostin to angiogenesis as the survival-promoting signaling pathway was also activated in endothelial cells (Figure 5) (7). This is in accordance with the increased occurrence of tumor blood vessel in metastases of periostin-overexpressing 293T cells (77).

Oskarsson et al. revealed an inverse time course of the role of tumor-endogenous and stromal tenascin C. While an induced knockdown of the protein diminished metastatic engraftment in the beginning, tumor cells induced myofibroblasts to produce tenascin C when the tumors reached a certain size, compensating the loss of the matricellular protein. This was consistent with immunohistochemical staining of human breast cancer samples, revealing tumor cell tenascin C expression in early malignant stages, and a strong reaction in stromal cells in advanced cirrhotic carcinomas (17). As a mechanism, Oskarsson et al. suggested the augmentation of signaling pathways, ensuring tumor cell survival at the unpermissive site, namely WNT and NOTCH signaling (Figure 5). Although having its main function in creating a metastatic niche and inducing WNT and NOTCH signaling pathways, tenascin C depletion did not correlate with a reduction of stem cell characteristics of the breast cancer cells in this study (23). These results are supported by another study in the 4T1 orthotopic model of breast cancer, where S100A4^+^ stromal cells were found to promote metastatic colonization to the lungs by producing high levels of tenascin C. In addition, tail vein-injected 4T1 cells showed a significantly lower efficiency of final engraftment in the lungs in tenascin C knockout mice (120).

## Rationale of Targeting the ECM

As metastases rather than the primary tumor cause the poor prognosis of most cancer patients, it is clearly important to stop the dissemination of cancer to and growth at distant sites of the body. Targeting the ECM, often secreted by stromal cells, is hereby of particular interest as these targets are less prone to rapid mutation as seen for signaling pathways in cancer cells. In addition, combinatorial therapies, integrating standard chemotherapy with ECM targeting, are an important strategy of cancer treatment in the adjuvant setting. Here, the hope is that the disruption of the dense tumor microenvironment will promote drug delivery and prevent chemoresistance. Although former studies concentrate on enzymes as LOXs or proteases like the MMPs responsible for remodeling ECM, here we focus on studies about targeting key ECM proteins.

## Targeting HA

### HA Deposition and Synthesis

The HA-rich pericellular matrix around cancer cells can be an obstacle for monoclonal antibody therapy, as the NK cells mediating the antibody-dependent cell-mediated cytolysis (ADCC) cannot come in close enough contact with the cancer cell to form the cytolytic synapse (98). Dissolving this protective halo *in vitro* through a PEGylated recombinant human hyaluronidase PH20 (PEGPH20) increased ADCC, and *in vivo* the combined treatment of HA-overexpressing SKOV3 ovarian cancer xenografts with PEGPH20, trastuzumab, and NK cells resulted in a drastic inhibition of tumor growth (98).

Besides sensitizing cancer cells to ADCC, hyaluronidase treatment of cancer also affects the biomechanics of HA-rich tumors. Accumulation of HA increases the colloid osmotic pressure leading to an increase in IFP within the tumor, causing multiple problems for treatment (121). An increase in IFP results in decreased transcapillary transport of solutes into the tumor, including systemic treatment. The increased IFP can also cause unstable tumor vessels to collapse, cutting off perfusion to areas of the tumor, creating hypoxia and further limiting tumor penetration of any systemic treatment. Accordingly, treating tumors with hyaluronidase lowers the IFP and increases the perfusion of tumors and the penetration of therapeutics (27, 122–124).

Although hyaluronidase treatment of cancers produces LMW-HA fragments, which can increase angiogenesis and disrupt vessel integrity, PEGPH20 treatment of experimental pancreatic cancer in combination with chemotherapy increased overall survival (27, 124) and decreased the metastatic incidence (124). It is reassuring to see that the creation of more functional vessels did not lead to an increase in metastasis, as it might have been feared to do. It is conceivable that by stripping cancer cells of their pericellular matrix, the hyaluronidase treatment is sensitizing any extravasating cancer cells to immune cell lysis, in this way counteracting the effect of restoring tumor vessel function. PEGPH20 is now in a randomized phase 2 clinical trial for metastatic pancreatic cancer and shows promising results (125). However, the clinical trial also revealed a serious side effect of the treatment, as the incidence of thromboemboli increased in the PEGPH20 treatment arm. Fortunately, this appears to be preventable by giving the patients the prophylaxis enoxoparin, and now a large, randomized, double-blinded, placebo-controlled phase 3 study is planned to start in the beginning of 2016 (125).

Instead of breaking down already produced HMW-HA, another option is to prevent its synthesis. 4-Methylumbelliferone (4-MU) is a cumarin derivative that inhibits the production of HA, most likely by depleting the cell of the UDP-glucuronic acid that is a precursor of HA (126). 4-MU is a naturally occurring compound, and it is has been approved in Europe since the 1960s to treat biliary spasms, thus repurposing it for cancer treatment is a very real possibility. Several *in vivo* studies have reported promising inhibition of tumor growth and metastasis formation upon treatment with 4-MU [reviewed in Ref. (127)]; however, so far no clinical trials or safety studies aimed at evaluating long-term safety have been initiated.

### Using HA as a Tag to Target Drugs to Cells

Many malignancies express CD44, thus using HA as a homing missile for cancer therapeutics is a logical step. Coupling of nanoparticles loaded with the chemotherapeutic drug paclitaxel to ultrashort HA polymers leads to improved drug uptake in brain tumor lesions of breast cancer in a preclinical model of brain metastases, significantly improving the overall survival of the mice treated with the nanoconjugate (128). Another study found that liposomes coated with PEG and HMW-HA had increased cellular uptake and tumor penetration in subcutaneous MDA-MB-231 tumors, even though the overall accumulation in the tumor was the same as PEG liposomes (129). Loading HA-coated liposomes with doxorubicin had an increased therapeutic effect in multiple tumor models, including experimental B16F10 melanoma metastases in the lungs (130).

One caveat of using HA as a tag to target chemotherapy to CD44^+^ cancer cells is the abundant expression of the HA receptor HARE/Stab2 in the liver, thus special attention to liver toxicity is essential in such studies. One way of limiting potential liver toxicity could be to try to exhaust the endocytic HARE/Stab2 presence on the hepatocytes by increasing the levels of HA in the blood before administering the HA–drug conjugate. Such an approach might even have the added benefit of preventing circulating tumor cells from attaching to endothelial cells and seeding new organs (104, 105), and elevated blood HA levels have shown no adverse effects in mice (105).

### Small Oligo-HA as Direct Treatment

Small oligo-HA (sHA) has been tested in multiple pre-clinical studies following the rationale that it can prevent the normal signaling from endogenous, often larger, HA molecules by occupying the HA binding partners/receptors. Indeed, inhibition of tumor growth *in vivo* in breast cancer, lung cancer, osteosarcoma, and melanoma has been reported. The addition of sHA prevents the formation of the versican–HA-rich pericellular matrix (36, 38) and inhibits the binding of ovarian cancer cells to the peritoneal wall *in vivo* (38). Most interestingly, the direct injections of sHA into tibial tumors of MDA-MB-231 breast cancer cells reduced the progression of already established osteolytic lesions (119). However, sHA fragments of a similar size correlate to increased lymphatic invasion and development of lymph node metastasis in colorectal cancer patients, suggesting that small sHA may promote tumor progression (131). The conflicting reports of the pro- and anti-tumorigenic effects of sHA are possibly due to a combination of differences in both size and concentration. Low concentrations of sHA stimulated the neovascularization of Matrigel plugs *in vivo* while very high concentrations inhibited angiogenesis (53). Thus, further studies with sHA should test a range of different sizes and concentrations to evaluate whether there is a large enough therapeutic window regarding bioavailability in the tumor that could make the treatment translational.

## Targeting Periostin

One example is the monoclonal periostin-blocking antibody OC-20. Although Orecchia et al. only reported beneficial aspects of the antibody on the primary tumor in their mouse model of human melanomas (132), the monoclonal antibody could be utilized in further preclinical studies investigating its influence on metastatic spread. The combination of a periostin-blocking antibody and 5-fluorouracil enhanced the apoptotic effect of the pyrimidine analog *in vitro* (133).

A further approach to target periostin is the benzyl-d(U)TP-modified nucleic acid aptamer PNDA-3. PNDA-3 specifically antagonized periostin-induced adhesion and invasion of breast cancer cells. In the 4T1 orthotopic mouse model, intratumoral PNDA-3 administration significantly reduced the size of the primary tumor and, more interestingly, of metastatic foci in the lung. The aptamer abrogated AKT/PKB pathway-mediated survival at the metastatic sites (134). PNDA-3 also reduced the metastatic burden of gastric cancer cells in the liver (135). Taken together, the DNA aptamer PNDA-3 is a promising drug for targeting periostin. New approaches combining nucleic acid aptamers with nanoparticle leading to a prolonged stability and specific binding to the tumor tissue could further strengthen the beneficial effect of the treatment qualifying the drug for clinical trials.

## Targeting Tenascin C

The large splice variant of tenascin C is specifically expressed in tumors and expression levels increase with grade of malignancy in many cancer types including brain, lung, and squamous cell cancer (21, 136). Therefore, targeting tenascin C is of particular interest for diagnostic and therapeutic approaches. Several (pre-)clinical studies using tenascin C-targeting agents were performed, in particular in advanced brain tumors such as glioblastoma multiforme and malignant astrocytomas.

First, a murine anti-tenascin monoclonal antibody named 81C6 was shown to bind specifically to tumor tissue (137). Neuradiab, the (131)I-labeled 81C6, showed decent benefits in a glioblastoma xenograft mouse models (138). Phase I and II studies where the antibody was injected directly into the resection cavity of glioblastoma patients revealed an improvement in the median survival of the patients (139–141); however, phase III trials were suspended owing to a delay in site initiation (clinicaltrials.gov).

To overcome the restriction of a non-recurrent and local administration of the murine IgG2, several human antibodies targeting tenascin C were generated. Silacci et al. developed a tenascin C-targeting antibody, named G11. The human monoclonal antibody stained patient-derived tumor sections with high specificity confirming the restricted expression of tenascin C to cancerous tissue. In addition, biodistribution studies in either mice bearing subcutaneous human glioma cell tumors or rats with orthotopic brain tumors, showed a highly selective tumor uptake of the antibody (136). In addition, Brack et al. developed a 125I-labeled humanized antibody targeting tenascin C, called F16. In a xenograft model, this radiolabeled human IgE antibody showed a selective targeting to human glioblastoma tissue with a clearance from the blood and other organs within 24h after intravenous injection (142). Both antibodies are promising approaches for further clinical trials, allowing a repeated treatment of cancer patients.

In addition, Kim et al. developed a tenascin C-targeting peptide binding specifically to the large splice variant of the protein either in a xenograft model of glioblastoma or in human lung and colon cancer or squamous carcinoma samples. This peptide reduced the migration of a glioblastoma cell line *in vitro* (143). Coupling this peptide to chemotherapeutics or radioactive isotopes could improve the clinical outcome of many cancer types.

Another way to target tenascin C is RNAi. When ATN-RNA, a double-stranded RNA complementary to human tenascin C, was applied locally to patients with advance brain tumors, the drug showed a survival benefit of 18 weeks in grade III astrocytoma and 10 weeks in glioblastoma multiforme (144). Thus, targeting tenascin C particularly for brain cancers looks very promising.

## Targeting Collagen I

Collagen prolyl-4-hydroxylases (CP4H) are essential enzymes for the correct biosynthesis of collagens. CP4H catalyzes the conversion of proline to hydroxyproline, facilitating the assembly of three procollagen proteins into a procollagen triple helix. Recent studies in preclinical breast cancer models have shown that inhibition of CP4H either completely prevents or dramatically decreases spontaneous metastasis to the lungs (65, 66).

Interfering with post-translational cross-linking of collagens by LOX-family members has been proposed as a promising therapeutic avenue due to successful inhibition of metastasis in pre-clinical models (43, 110). So far, a LOXL2-targeting antibody (145) is in clinical trials for the treatment of fibrosis and cancer, but recently phase II clinical trials combining this antibody with chemotherapy (gemcitabine) in pancreatic cancer did not yield positive results (Gilead, press release). However, recent preclinical data have shown efficacy of targeting LOX in combination with gemcitabine in preclinical models of pancreatic cancer, provided treatment was administered early (146). Thus, LOXL2 inhibition combined with gemcitabine could still be effective in early-stage patients.

## Concluding Remarks and Future Approaches

The ECM has crucial impact on cancer progression. In this review, we highlighted how ECM proteins influence every step of the complex cascade of tumor spread with a focus on some of the key players (Figure 1). The ECM not only provides cues favoring migration, attachment, survival, or proliferation but also physically protects the cells from the shear stress in the blood, immune cell attack, and the impact of drugs. Targeting ECM proteins is therefore of great interest in the adjuvant therapeutic setting. In particular, combination treatment with gold-standard therapeutics seems to be a promising strategy, as targeting the macromolecules facilitates the delivery and efficacy of the standard treatment and inhibits protumorigenic signaling of the ECM itself.

Extracellular matrix proteins can be targeted in many different ways. Beyond the direct targeting of the macromolecules, one can also prevent their synthesis, cross-linking, or correct post-translational processing, opening up many possible avenues of treatment. However, although targeting of ECM components holds much promise for improved cancer management, it is important to keep in mind that any treatment aimed at cancer ECM also may affect the same ECM component in healthy tissue. Indeed, one of the main reasons why the much anticipated MMP inhibitors had such a poor success rate in clinical trials was the unexpected toxicity in healthy tissue [this reason and others are reviewed in Ref. (147)]. It is thus crucial to develop and rigorously study preclinical models of ECM targeting in many cancer types, paying particular attention to any side effects in healthy tissue to overcome this translational hurdle and move from bench to bedside. Additionally, further research into the exact mechanisms of how the individual ECM macromolecules stimulate migration, adhesion, and cell survival, and how this may be different in cancerous contra normal settings, will help to tailor targeting strategies to suit cancer ECM better than healthy ECM. Another important lesson that can be learned from the initial failure of MMP inhibitors is about the timing of the treatment with regard to the cancer progression stage. The ECM components reviewed here often play a part in more than one of the steps of the metastatic cascade, but the involvement in one step might turn out to be more targetable than others. This makes it paramount for preclinical studies to carefully dissect when a given target should be hit to have an effect and, in effect, making it very important to stratify clinical trials accordingly.

Nevertheless, targeting components of the ECM in order to block cancer-driving signaling pathways and to facilitate the penetrance of standard chemotherapeutics is a promising approach to prevent the life-threatening spread of cancer.

## Conflict of Interest Statement

The authors declare that the research was conducted in the absence of any commercial or financial relationships that could be construed as a potential conflict of interest.
